# Nitride–Silver Hybrid PCF-SPR Biosensor: A High-Sensitivity Platform for Synchronous Monitoring of Low-Concentration Analytes and Temperature

**DOI:** 10.3390/s25175292

**Published:** 2025-08-26

**Authors:** Chenyu Liang, Junzhu Wang, Jiaxuan Zhu, Jie Zhao, Kai Zhang

**Affiliations:** College of Metrology Measurement and Instrument, China Jiliang University, Hangzhou 310018, China

**Keywords:** photonic crystal fiber, surface plasmon resonance, dual-parameter sensing, biosensor, sensitivity enhancement

## Abstract

This study proposes a dual-parameter photonic crystal fiber-based surface plasmon resonance (SPR) sensor for simultaneous refractive index and temperature detection. The sensor architecture incorporates an asymmetric air hole lattice, featuring elliptical inner holes (aspect ratio: 1.5) to enhance birefringence and axially aligned outer circular holes to optimize surface plasmon coupling. Horizontally, symmetrically deposited silver films and silicon nitride layers constitute the RI-sensing channel, while a vertically machined PDMS-coated silver–nitride structure enables temperature responsivity. The temperature-sensing channel delivers a sensitivity of 20 nm/°C within 0–100 °C, while the RI channel achieves a peak sensitivity of 18,600 nm/RIU across *n_a_* = 1.33–1.41 with a resolution of 5.38 × 10^−6^ RIU. Notably, cross-sensitivity between the two channels remains below 5%, underscoring the sensor’s capability for independent dual-parameter analysis. This low-interference, high-sensitivity platform holds significant promise for advanced biosensing applications requiring real-time multiparametric monitoring.

## 1. Introduction

Photonic crystal fiber (PCF) represents a novel category of optical fibers, distinguished by the presence of two-dimensional periodic arrays of pores or doped regions along their length within a pure silicon matrix. In accordance with the photoconductive mechanism, the primary classification of PCFs is into two distinct types: index-guided and bandgap-guided [1]. Bandgap-guided PCFs facilitate the transmission of light at specific wavelengths through the photonic bandgap effect. However, refractive index-guided PCFs operate in a manner analogous to conventional optical fibers, with their functionality being contingent on the refractive index contrast between a silica core and a microstructured cladding consisting of air holes. The optical properties of PCFs, including dispersion, nonlinearity, and birefringence, can be meticulously tailored by means of adjusting the structural parameters such as air hole diameter, spacing, and lattice symmetry [2,3].

PCF exhibits a number of advantageous properties, including high birefringence, design flexibility, low loss, wide bandwidth, high nonlinearity, and sensitivity to environmental changes. These properties make it an ideal platform for integrating surface plasmon resonance (SPR) technology [4]. The physical phenomenon of SPR occurs due to the interaction of evanescent waves and free electrons in metallic nanostructures, resulting in a resonant coupling, which provides a new solution for the real-time detection of micro-volumes. PCF-SPR sensors exploit the augmented light–matter interactions within the PCF, thereby delivering a revolutionary solution for the detection of ultra-low concentrations of biomolecules (e.g., proteins, DNA) in the domain of biosensing.

The optimization of PCF structural parameters (e.g., pore geometry, lattice arrangement, and metal layer composition) enables the simultaneous high-sensitivity detection of multiple physical parameters. In the scientific realm, the precise and concurrent measurement of the refractive index (RI) and temperature is of paramount importance across numerous domains of research, including environmental monitoring, biomedical diagnostics, and industrial process control [5,6,7,8]. Real-time monitoring of these parameters has been demonstrated to enhance the precision of environmental testing, production process control, and early disease detection. However, due to inherent cross-sensitivity limitations, conventional sensors often struggle to achieve high sensitivity and selectivity for two-parameter detection. This challenge necessitates the development of advanced sensing platforms that can effectively decouple and quantify refractive index and temperature independently and with reduced interference. PCF-SPR sensors have emerged as a key solution to address these limitations. The multiplexing of the sensor for the sensing and independent quantification of multiple analytes is enabled by optimizing the PCF structural parameters, such as adjusting the pore geometry, lattice arrangement, and metal layer composition, thereby reducing cross-talk.

The enhancement of sensitivity and the augmentation of the detection range remain pivotal objectives in the research of optimizing the performance of SPR sensors. Rupam Srivastava et al. [9] developed a sensor of the PCF-SPR variety that is characterized by a double-sided polishing process with a semicircular groove for the detection of a variety of analytes, including water, plasma, white blood cells (WBCs), hemoglobin (HB), and red blood cells (RBCs), over a wide range of RIs (1.17–1.40). The semicircular notch design has been shown to minimize the core-to-analyte distance, thereby achieving a maximum wavelength sensitivity of 13,000 nm/RIU and a resolution of 7.7 × 10^−6^ RIU. The dual-core structure and multi-polishing configuration further facilitate multiparameter detection or multifunctional integration. Ahmed Oudenani et al. [10] put forward the proposal of an SPR-based D-type PCF sensor, which would incorporate a central aperture that would be filled with liquid crystals for the purpose of simultaneous temperature and RI detection. The design exhibits an RI sensitivity of 17,900 nm/RIU (range: 1.42–1.51), an amplitude sensitivity of 557.60 RIU^−1^, and a resolution of 5.58 × 10^−6^ RIU. Concurrently, it demonstrates a temperature sensitivity of 4.2 nm/°C (15–35 °C) and a resolution of 0.023 °C. The dual-parameter detection capability under discussion is indicative of its potential for use in biomedical diagnostics and environmental monitoring, particularly in scenarios where simultaneous refractive index and temperature analyses are required.

The adoption of novel materials and nanostructured architectures, such as discontinuous metallic coatings and nanoparticle-based configurations, offers innovative ways to advance PCF-SPR sensor performance. Farhan Mumtaz et al. [11] achieved notable enhancement by uniformly depositing MXene nanosheets (Ti3C2Tx) onto fiber grooves, leveraging their exceptional electrical conductivity and high surface-to-volume ratio. In a complementary approach, Yanxin Yang et al. [12] developed a polydimethylsiloxane (PDMS)-integrated PCF sensor that incorporates size-tunable gold nanoparticles (AuNPs). By optimizing the AuNP diameters, they maximized the coupling of localized surface plasmon resonance (LSPR), achieving a dual-channel sensor with peak wavelength sensitivity of 13,000 nm/RIU and a resolution of 7.69 × 10^−6^ RIU. These innovations not only address oxidation-induced performance degradation but also enable spectral customization for analyte-specific detection in complex biological environments.

Despite significant advances in PCF-SPR sensors, there are still technical challenges in terms of manufacturing accuracy, sensitivity, long-term stability, and cost-effectiveness. For example, non-uniformity in the thickness of the metal coating can lead to inconsistent resonance peak shifts, reducing the repeatability of measurements. For instance, a 2 nm deviation in the thickness of the gold coating results in a 10–15 nm wavelength shift [13], significantly impacting sensor consistency. Secondly, the oxidation of the metal layer (e.g., silver oxidation in humid environments) reduces plasmonic coupling efficiency and causes problems with long-term stability [14]. Thirdly, multilayer coating structures enhance functionality but increase manufacturing cost and complexity [15]. Finally, multiparameter sensing systems face inherent signal crosstalk problems [16,17], limiting their utility in sophisticated applications such as biomarker detection and simultaneous temperature monitoring.

This study presents a novel PCF-SPR sensor structure that has been optimized for the simultaneous detection of the refractive index and temperature. The design incorporates a multi-channel PCF structure and a bifunctional plasmonic coating to improve sensitivity and selectivity by optimizing geometric parameters and innovating materials. Specifically, silver was used as the plasmonic layer for RI-sensing, polydimethylsiloxane as the thermal medium for temperature detection, and a silicon nitride (Si_3_N_4_) buffer layer to minimize interfacial losses. Conventional methods requiring the deposition of metal within air holes are complex to manufacture, particularly with regard to ensuring uniform coating thickness and alignment accuracy—issues that hinder scaling up. The issue previously mentioned in this text is dealt with by the present design, which is able to position the plasmonic layer on the outer surface of the PCF. This, in turn, greatly simplifies the fabrication process. Taking advantage of the structural asymmetry, the orthogonal polarization modes are decoupled into different RI and temperature responses, thereby suppressing crosstalk to below 5% and enabling independent modulation of the resonant peaks. Finite element method (FEM) simulations validate sensor performance by analyzing mode coupling efficiency, confinement loss, and resonance wavelength shift for different refractive index (1.33–1.41) and temperature (0–100 °C) conditions.

This work advances the theoretical understanding of the two-parameter sensing mechanism in the PCF-SPR system and provides a scalable framework for developing high-performance sensors for biochemical analysis, environmental monitoring, and industrial automation.

## 2. Theoretical Foundations and Structural Design

**Figure 1:**
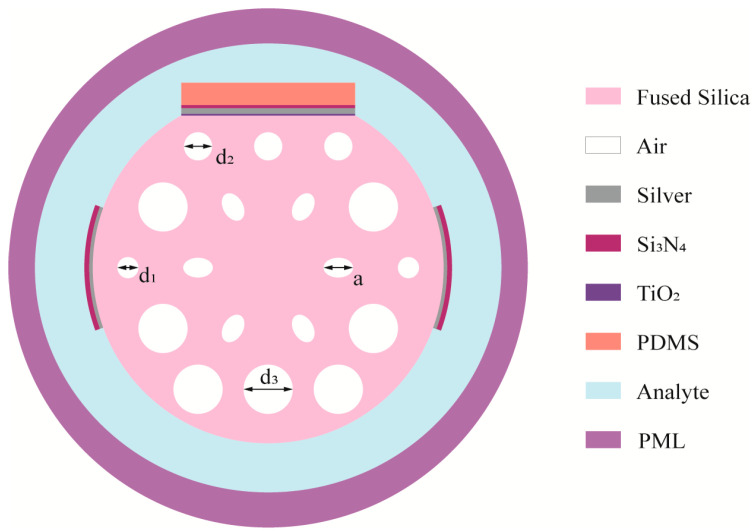
Schematics of the proposed structures.

As illustrated schematically in Figure 1, a two-parameter PCF sensor is based on the surface plasmon resonance effect. The thickness of the plasmon excitation layer was initially set to 40 nm, as the range of 30–50 nm has been shown to balance sufficient plasmon excitation with field coupling. It has been established that films with a thickness of less than 25 nm result in insufficient surface plasmon localization. Conversely, films with a thickness greater than 55 nm cause the plasmon mode to deviate to such an extent from the core that there is a reduction in both overlap and sensitivity. Subsequently, through a combination of parameter scanning and finite element modal analysis, the remaining layer parameters were optimized, resulting in the following design scheme. A silver film with a thickness of t1 = 35 nm was symmetrically deposited on both sides of the sensor in the horizontal direction. A silicon nitride layer of the same thickness (t2 = 40 nm) was then deposited on top of the silver film to enhance the SPR excitation intensity within the target refractive index detection range. When viewed vertically, the top of the sensor has a machined plane located h =4.46 um from the center. On this plane, a layer of titanium dioxide with a thickness of t3 = 10 nm is first deposited, followed by a silver film with a thickness of t4 = 30 nm. Subsequently, a silicon nitride layer with a thickness of t5 = 30 nm was then deposited on the silver film. Finally, a thermally responsive PDMS layer was spin-coated onto the metal surface after a parametric sweep (0.7–2 µm) that revealed the optimal thickness to be 1 µm. Within this window, thinner films attenuate the thermal response while thicker ones prolong diffusion; therefore, the chosen 1 µm value secures both rapid equilibration and maximal temperature-induced resonance shift, thereby enabling reliable dual-parameter sensing of refractive index and temperature.

It is evident that the PCF structure comprises two concentric layers of perforations, with a uniform pitch of Λ = 2 µm across 18 holes. The inner air holes are designed as elliptical with an aspect ratio of 1.5, featuring major axes of a = 0.48 µm, thereby optimizing the modal field distribution of x- and y-polarized light. The outer layer incorporates circular air holes of three distinct radii (d1 = 1.4 µm, d2 = 0.8 µm, d3 = 0.6 µm), with smaller holes preferentially aligned along the axial direction to facilitate efficient energy exchange between the SPP layer and the fiber core.

The material utilized as background for PCF is pure silica. The dispersion properties of pure silica can be accurately determined by Equation (1) [18]:

(1) $$n_{silica}={\left(\begin{array}{l} 1.031552+0.690754\times 10^{-5}T+\frac{(0.788404+0.235835\times 10^{-6}T)\lambda^{2}}{\lambda^{2}-(0.0110199+0.584758\times 10^{-6}T)}\\ +\frac{(0.91316+0.548368\times 10^{-6}T)\lambda^{2}}{\lambda^{2}-100\;} \end{array}\right)}^{\frac{1}{2}}$$

Here, *n_silica_* represents the refractive index of silica, *λ* denotes the wavelength of light in micrometers (um), and *T* indicates the temperature in degrees centigrade.

The SPR is to be stimulated at the interface of the silver layer. The complex dielectric constant of the copper film can be described by the Drude–Lorentz model and written as follows [19]:

(2) $$\varepsilon_{m}\left(\lambda \right)=\varepsilon_{mr}+\varepsilon_{mi}=1-\frac{\lambda^{2}\lambda_{c}}{\lambda_{p}^{2}\left(\lambda_{c}+i\lambda \right)}$$

where *λ_c_* and *λ_p_* are the collision wavelength and plasma wavelengths of the metal layer, respectively. For silver, *λ_c_* = 1.76 × 10^−5^ m and *λ_p_*= 1.454 × 10^−7^ m [20].

The refractive index of silicon nitride (Si_3_N_4_) with respect to wavelength can be calculated using the Sellmeier dispersion equation [21]:

(3) $$n_{{\mathrm{Si}}_{3}N_{4}}={\left(1+\frac{\left(3.0249\lambda^{2}\right)}{\left(\lambda^{2}-0.1353406^{2}\right)}+\frac{\left(40314\lambda^{2}\right)}{\left(\lambda^{2}-1239.84^{2}\right)}\right)}^{\frac{1}{2}}$$

where *λ* is the wavelength in microns (μm). The refractive index model exhibits very high accuracy in the 1550 nm band, with an error rate of less than 0.5%.

The titanium dioxide (TiO_2_) interlayer, which is deposited between the metal film and the polished fiber surface, serves as a reinforcement layer for adhesion, significantly improving the bond strength. This prevents delamination-induced degradation of the metal coating, thus ensuring the long-term stability of the sensor. The refractive index of this layer can be calculated using the following formula:

(4) $$n_{{\mathrm{TiO}}_{2}}={\left(5.913+\frac{0.2441}{\lambda^{2}-0.0803}\right)}^{\frac{1}{2}}$$

Polydimethylsiloxane (PDMS) has a significant negative thermo-optic coefficient (TOC) of approximately −4.5 × 10^−4^ RIU/°C [22], resulting in a substantial variation in its refractive index across different temperatures. This makes PDMS highly sensitive to temperature changes and an ideal material for high-performance temperature sensors. In contrast, the TOC of silicon dioxide (SiO_2_) is much smaller at around −6 × 10^−6^ RIU/°C [23] and is usually considered negligible in temperature-related calculations. The relationship between the refractive index of PDMS and the prevailing temperature can be expressed through the following Equation (5):

(5) $$n_{PDMS}=-4.5\times 10^{-5}\cdot T+1.4176$$

where *T* is the temperature in degrees centigrade.

The outermost layer of the sensor is the analyte layer, which has a refractive index of na. To improve the accuracy of the simulation, a Perfectly Matched Layer (PML) has been added to the finite element method (FEM) model based on COMSOL Multiphysics 6.3 outside the analyte layer to efficiently absorb radiant energy at various angles of incidence. This configuration prevents interference with the energy distribution in the fiber. The modal analysis is performed in the X-Y plane while light propagates along the Z direction.

Performance Parameters

The limited loss of the fiber optic transmission mode is a pivotal parameter in characterizing the performance of surface plasmon resonance sensors. It has been demonstrated that there is a direct correlation between the confinement loss and the imaginary component of the effective refractive index. This relationship is mathematically expressed as [24]:

(6) αdB/cm=8.686×k0×Imneff×104

In the context of electromagnetic theory, the wavelength in free space, denoted by *k*_0_, is defined as the ratio of the wave’s angular frequency to its wavelength. The imaginary part of the effective refractive index, denoted by Im(*n_eff_*), is a measure of the wave’s refractive properties.

The wavelength sensitivity is calculated using the wavelength interrogation method. It is generally accepted that the wavelength interrogation technique is more sensitive than the amplitude interrogation technique. The following equations [25] mathematically express the relationship between wavelength shift and changes in the surrounding refractive index and temperature:

(7) $$S_{\lambda }\left(\mathrm{nm}/\mathrm{RIU}\right)=\frac{\Delta \lambda_{peak}}{\Delta n_{a}}$$

(8) $$S_{\lambda }\left({}^{\circ}C/\mathrm{RIU}\right)=\frac{\Delta \lambda_{peak}}{\Delta T}$$

Here, the change in resonance wavelength is denoted by Δ*λ_peak_*, the difference between two neighboring refractive indices is denoted by Δ*n_a_*, and the change in temperature is denoted by Δ*T*.

The resolution (*R*) of a sensor is defined as the smallest detectable change in the target parameter. Recent studies have demonstrated the capacity of high-resolution sensors to accurately measure and recognize minute parameter changes. The resolution of the RI- and temperature-sensing channels of the proposed two-parameter photonic crystal fiber surface plasmon resonance sensor is calculated as follows [26]:

(9) $$R_{RI}\left(\mathrm{RIU}\right)=\frac{\Delta n_{a}\times \Delta \lambda_{\min}}{\Delta \lambda_{peak}}$$

(10) $$R_{T}\left({}^{\circ}C\right)=\frac{\Delta T\times \Delta \lambda_{\min}}{\Delta \lambda_{peak}}$$

where *R_RI_* and *R_T_* represent the resolutions for *RI*- and temperature-sensing, respectively. The symbol Δ*λ*_min_ denotes the minimum wavelength offset that can be resolved, and it is set to 0.1 nm. Furthermore, the Δ*λ_peak_* is employed to denote the wavelength difference between two adjacent resonance peaks.

Amplitude sensitivity is defined as the ratio of the change in amplitude of the sensor output signal to the change in the measured parameter and is an important performance indicator of the sensor system. For the purpose of calculating the amplitude sensitivity for refractive index- and temperature-sensing channels, the following expression may be utilized [27]:

(11) $$S_{A_{RI}}\left({\mathrm{RIU}}^{-1}\right)=-\frac{1}{\alpha \left(\lambda ,n_{a}\right)}\cdot \frac{\partial \left(\lambda ,n_{a}\right)}{\partial n_{a}}$$

(12) $$S_{A_{T}}\left({\mathrm{RIU}}^{-1}\right)=-\frac{1}{\alpha \left(\lambda ,T\right)}\cdot \frac{\partial \left(\lambda ,T\right)}{\partial T}$$

In this equation, *α*(*λ*,*n_a_*) denotes the confinement loss function when the analyte’s RI is na, and *∂*(*λ*,*n_a_*) is the minute variation in the confinement loss function due to a slight change in analyte RI. Similarly, *α*(*λ*,*T*) refers to the confinement loss function when the analyte’s temperature is designated as *T*, and *∂*(*λ*,*T*) is the function’s variation from a minor temperature variation.

The performance of a sensor is determined by a number of factors in addition to its sensitivity. These include the full width at half maximum (FWHM), figure of merit (FOM), and crosstalk levels. A narrower FWHM indicates a sharper resonance peak and higher spectral resolution. The figure of merit, FOM, is defined as the ratio of sensitivity to FWHM. Crosstalk is quantified by the ratio of response changes caused by interference factors to those caused by the target variable. This directly reflects the independence of each channel when multiple parameters coexist. Collectively, these three factors constitute a comprehensive and consistent evaluation framework for sensor performance.

## 3. Simulation Result Analysis and Discussion

**Figure 2:**
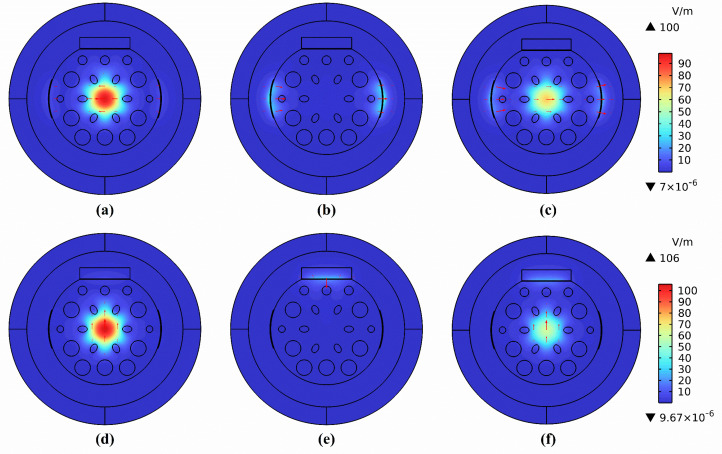
The mode field distribution: (**a**) x—polarized core mode at 1050 nm; (**b**) x—polarized SPP mode at 1144 nm; (**c**) x—polarized coupling mode at 1144 nm; (**d**) y—polarized core mode at 850 nm; (**e**) y−polarized SPP mode at 1144 nm; (**f**) y—polarized coupling mode at 1144 nm.

Figure 2a–f systematically demonstrate the modal characteristics of the sensor under x- and y-polarized excitation, including the fundamental core modes, the localized surface plasmon polariton modes, and the coupled hybrid modes. The color scale for the top three images can be referenced from the color chart in image (c), while the color scale for the bottom three images can be referenced from the color chart in image (f). Specifically, Figure 2a–c illustrate the electric field distributions of the x-polarized core mode, the SPP mode, and their coupled hybrid modes, respectively. The energy of the core mode is confined within the silica fiber core by the cladding air holes, and the energy of the optical field in the SPP mode is concentrated at the interface between the PDMS and the analyte under test. Furthermore, as illustrated in Figure 2d,f, the corresponding y-polarization modes are demonstrated. The coupled hybrid modes manifest asymmetric energy localization, with the core mode transferring a proportion of its energy to the SPP mode through evanescent field coupling. The wavelength-dependence of this coupling mechanism is evident, with maximum overlap efficiency observed at the critical phase-matched wavelength (1170 nm). The distinct mode field distributions observed at varying polarization directions unequivocally substantiate the sensor’s pronounced birefringent properties and its exceptional two-parameter sensing capability.

**Figure 3:**
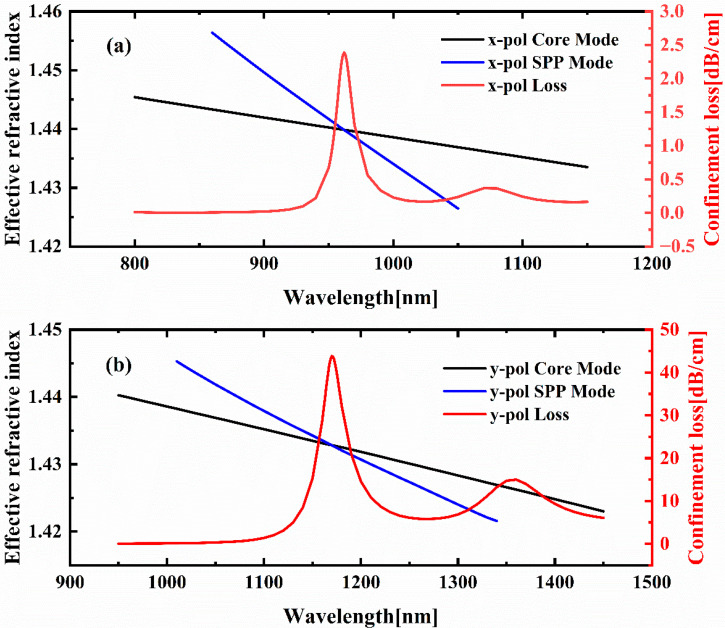
(**a**) The core mode, SPP mode, and confinement loss spectrum of the RI-sensing channel; (**b**) the core mode, SPP mode, and confinement loss spectrum of the temperature-sensing channel.

Figure 3a illustrates the dispersion relation between the x-polarized core mode and the surface plasmon polariton mode, along with the coupling characteristics between the y-polarized mode and the SPP mode. As demonstrated in Figure 3a, the black curve signifies the real part of the effective refractive index (Re(neff)) of the x-polarized core mode, whilst the blue curve corresponds to the Re(neff) of the SPP mode. As the incident wavelength increases, the black and blue curves intersect at a critical wavelength of 962 nm, indicating resonant coupling between the core and SPP modes. This phase-matching condition gives rise to a significant energy transfer between the two modes, resulting in a pronounced peak in the confinement loss spectrum of the x-polarized mode at this wavelength. In a similar manner, the black and blue curves in Figure 3b illustrate the Re(neff) with wavelengths for the y-polarized core mode and the SPP mode, respectively. At 1170 nm, the intersection of these curves indicates mode coupling accompanied by a maximum confinement loss for the y-polarized mode.

It is evident that alterations in the refractive index or temperature of the analyte have the capacity to modify the phase-matching conditions between the core and surface plasmon polariton (SPP) modes in the sensor. This, in turn, can result in variations in the resonance wavelengths and amplitudes of the x-polarized and y-polarized modes. Consequently, the sensor can be investigated by means of wavelength-probing and amplitude-probing methods.

**Figure 4:**
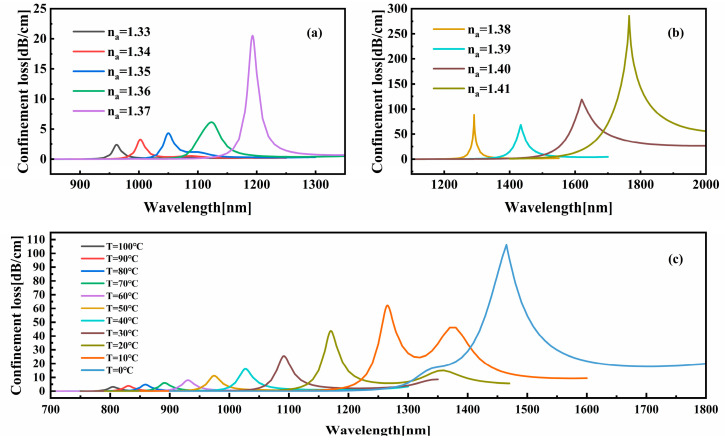
(**a**) Confinement loss plot of x-polarization when *n_a_* = 1.33–1.37. (**b**) Confinement loss plot of x-polarization when *n_a_* = 1.38–1.41. (**c**) Confinement loss plot of y-polarization on the analyte temperature.

In order to assess the RI sensitivity of the sensor, Figure 4 tracks the redshift of the x-polarization mode resonance wavelength as the RI(*n_a_*) of the analyte increases from 1.33 to 1.41. It has been demonstrated that as the refractive index of the analyte increases from 1.33 RIU to 1.41 RIU, characteristic changes in the transmission spectrum are exhibited, attributable to enhanced plasma coupling. When the RI of the analyte is between 1.36 and 1.37 RIU, a rapid increase in loss is observed with a sharp rise in the amplitude of the main loss peak. This phenomenon is attributed to the enhanced phase-matching between the core and SPP modes. At analyte RI values between 1.38 and 1.40 RIU, the resonance significantly broadens and the full width at half maximum (FWHM) increases due to mode decoupling at high RI values and increased radiative losses. This biphasic response thus confirms the ability of the sensor to discriminate subtle changes in RI by analyzing amplitude and spectral features.

The x-polarized resonance wavelength exhibits a linear redshift with rising *n_a_*, achieving a maximum sensitivity of 18,600 nm/RIU and a resolution of 5.376 × 10^−6^ RIU. The resonance intensity initially increases and then decreases with na, peaking at 1620 nm. As demonstrated in Figure 4c, the intensity of constraint loss exhibits a decline as the temperature rises from 0 °C to 100 °C with a resonance wavelength range of 1465 nm to 805 nm. The maximum recorded sensitivity is 23.8 nm/°C, with a resolution of 5.0 × 10^−3^ °C.

**Figure 5:**
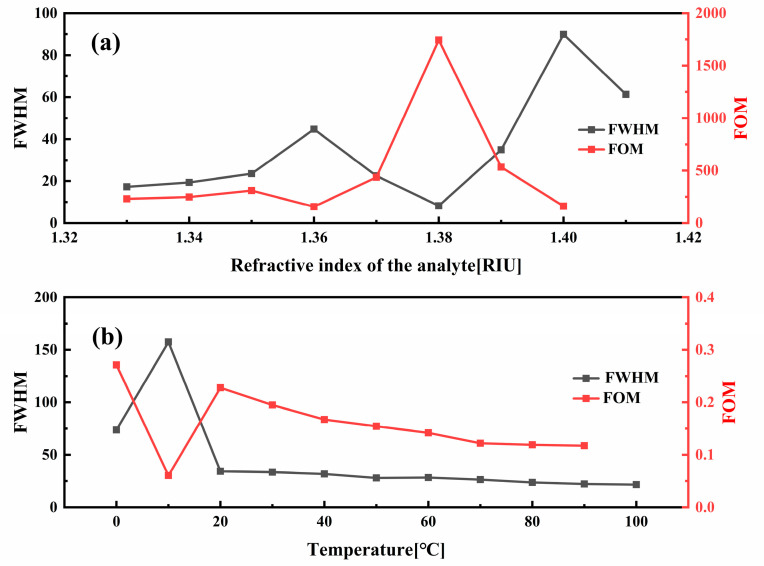
(**a**) FWHM and FOM values of the x—pol sense channel; (**b**) FWHM and FOM values of the y—pol sense channel.

As demonstrated in Figure 5a, the sensor demonstrates optimal FOM parameters when the refractive index of the analyte is approximately 1.38. As demonstrated in Figure 5b, the FOM value of the temperature-sensing channel exhibits a consistent decline with rising temperature, reaching a sudden decrease at 10 degrees Celsius. This phenomenon can be attributed to the interaction between a higher-order mode of the SPP mode and the core mode, which is also the underlying cause of the double peak observed in the constraint loss depicted in Figure 4c.

**Figure 6:**
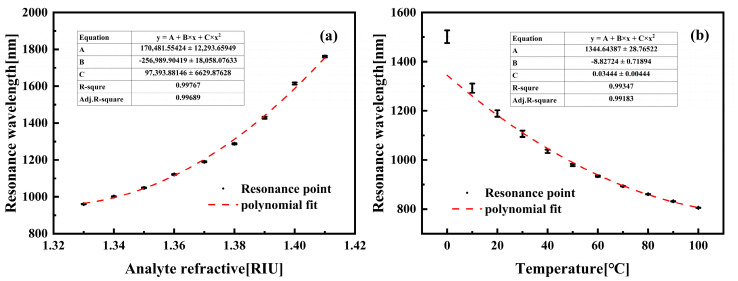
(**a**) Resonance wavelength of x—polarization on the analyte RI. (**b**) Resonance wavelength of y—polarization on the analyte temperature.

As shown in Figure 6a, the x-polarization resonance wavelength exhibits a direct linear relationship with the analyte refractive index. Within the detection range of *n_a_* = 1.33–1.42, the refractive index-sensing channel demonstrates enhanced linearity over the sub-range *n_a_* = 1.33–1.41, achieving a coefficient of determination of 0.99641. For the y-polarized temperature-sensing channel, increasing temperature reduces the PDMS layer’s refractive index, consequently inducing a blue shift in the y-polarized resonance wavelength. Figure 6b reveals a distinct inverse correlation between the y-polarized resonance wavelength and temperature. The linear response is notably stronger across the 0–100 °C range (R^2^ = 0.98524) than over the full operational temperature scope.

**Figure 7:**
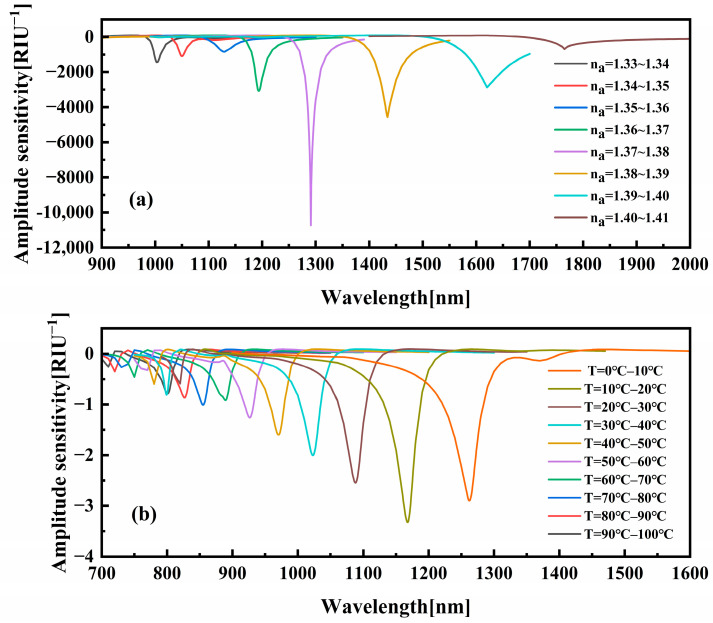
(**a**) Amplitude sensitivity response curve of the RI-sensing channel per 0.01 RIU; (**b**) amplitude sensitivity variation profile of the temperature-sensing channel at 10 °C intervals.

As illustrated in Figure 7a, the amplitude sensitivity curves vary with different RI at a constant temperature of 20 °C. The amplitude sensitivity curves are displayed in Figure 7a. As demonstrated in Figure 1, the amplitude sensitivities of the refractive index-sensing channel of the sensor at wavelengths of 1003, 1050, 1129, 1194, 1291, 1434, 1620, and 1765 nm are 1448.74, 1089.09, 841.91, 3282.4, 10,723.33, 4559.92, 2860.95, and 696.58 RIU^−1^, respectively. The maximum amplitude sensitivity at 1291 nm was determined to be 10,723.33 RIU^−1^, as the refractive index of the analyte increased from 1.37 to 1.38. As the refractive index of the analyte undergoes a change from 1.35 to 1.41, the amplitude sensitivity increases and subsequently decreases.

Figure 7b illustrates the variation in amplitude sensitivity demonstrated for distinct temperature intervals, while the refractive index of the analyte was maintained at 1.33. As demonstrated in Figure 1, the temperature-sensing channel of the sensor exhibits amplitude sensitivities at wavelengths of 801, 827, 855, 889, 926, 970, 1023, 1088, 1168, and 1262 nm, with a magnitude of 0.788, 0.864, 1.007, 0.916, 1.255, 1.597, 2.003, 2.545, 3.322, and 2.894 RIU^−1^, respectively. The maximum sensitivity at 1168 nm was determined to be 3.322 RIU^−1^ when the analyte temperature was increased from 10 °C to 20 °C.

### 3.1. Independence Analysis

In a two-parameter sensing system, the mutual independence of the two sensing parameters is of crucial importance. The independence of the detection channels is pivotal in ensuring the sensors accurately measure two distinct physical quantities without cross-talk. This independence is further crucial in ensuring that changes in one parameter do not affect the accuracy of the other.

**Figure 8:**
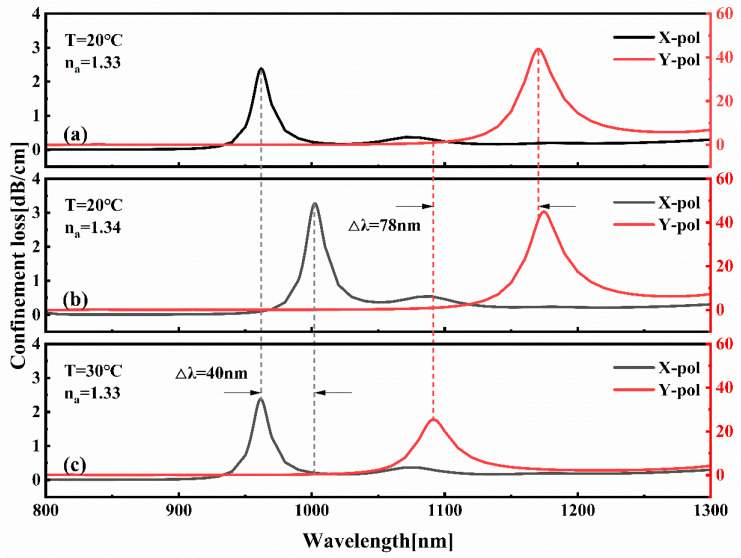
Loss curves of x- and y-polarization core modes: (**a**) *n****_a_*** = 1.33, T = 20 °C; (**b**) *n****_a_*** = 1.34, T = 20 °C; (**c**) *n****_a_*** = 1.33, T = 30 °C.

As illustrated in Figure 8, the constrained loss spectra of the x-polarized and y-polarized modes are examined under three distinct conditions: The following equations are to be used: T = 20 °C *n_a_* =1.33, T = 20 °C *n_a_* =1.34, and T = 30 °C *n_a_* =1.33. In these equations, T denotes the ambient temperature and *n_a_* denotes the refractive index of the analyte. As demonstrated in Figure 7b, upon increasing n from 1.33 to 1.34, the resonance peak of the x-polarization mode exhibits a redshift of 40 nm, while the position and intensity of the loss peak of the y-polarization mode remain predominantly constant. Conversely, at temperatures ranging from 20 °C to 30 °C (Figure 8c), a substantial shift of 78 nm is observed in the spectrum of the y-polarized mode. Concurrently, the resonance wavelength and coupling strength of the x-polarized mode (RI-sensing channel) exhibit remarkable stability.

**Figure 9:**
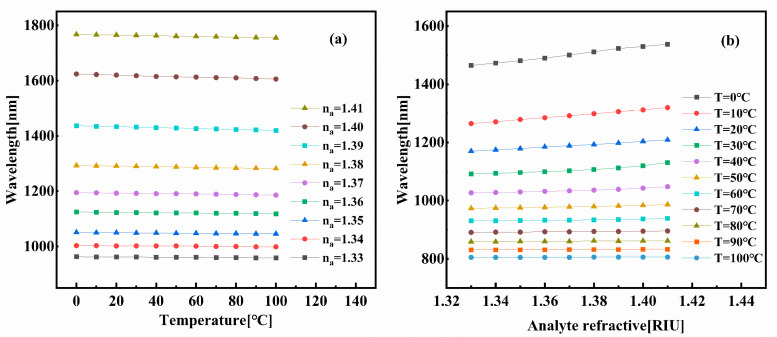
(**a**) Crosstalk generated by temperature changes in the refractive index-sensing channel. (**b**) Crosstalk generated by refractive index changes in the temperature-sensing channel.

As demonstrated in Figure 9, the scenario under consideration involves the impact of another variable on two sensor channels. As demonstrated in Figure 9, the refractive index sensor channel exhibits excellent stability and is less susceptible to temperature fluctuations. Conversely, the temperature sensor channel, particularly within the lower temperature range, demonstrates a substantial response to variations in refractive index. The crosstalk between the two channels has been measured at 0.23% and 13.83%, respectively.

The findings indicate a distinct independence between the two sensing channels, with x-polarization mode exhibiting a predominant sensitivity to alterations in refractive index and minimal susceptibility to temperature changes. Conversely, y-polarization mode demonstrates a heightened responsiveness to temperature variations and a lesser degree of sensitivity to changes in refractive index. This characteristic has been demonstrated to enhance the reliability and accuracy of the sensor’s two-parameter detection.

### 3.2. Performance Comparison

Table 1 and Table 2 present a comparative analysis of the sensing performance of the refractive index- and temperature-sensing channels of the proposed two-parameter PCF-SPR sensor with those of the same type of PCF-based advanced sensors. This analysis is conducted through a comprehensive comparison of sensitivity, resolution, and operating range, respectively.

Table 1 delineates the merits of the proposed refractive index-sensing design. Despite the sensor’s relatively narrow refractive index detection range, it exhibits exceptional sensitivity, particularly within the lower RI range. At *n_a_* = 1.40, the wavelength sensitivity reaches 18,600 nm/RIU, greatly exceeding that of comparable PCF-SPR sensors, which typically measure less than 15,000 nm/RIU in this range. This improved sensitivity is the result of asymmetric plasmonic coupling, which is due to optimized birefringence and elliptical aperture geometry. Furthermore, the sensor maintains a resolution of 5.376 × 10^−6^ RIU, which is more than an order of magnitude higher than that of conventionally designed SPR sensors. This performance makes it feasible for trace analytical detection in dilute biological solutions, where high sensitivity at low RI values is critical for analyzing samples.

Table 2 provides a comprehensive comparison of the temperature-sensing channel’s performance metrics, offering a detailed assessment of its characteristics relative to other similar sensors. The temperature-sensing channel strikes a balance between detection range and sensitivity. It covers a wide temperature range and excels in sensitivity. It can accurately capture subtle temperature variations within the detection range. This makes it ideal for supporting high-precision biochemical analyses.

### 3.3. Manufacturing Feasibility

In order to satisfy the stringent morphological requirements of the design, which stipulate the creation of inner elliptical pores with a major axis of 0.48 µm and an aspect ratio of 1.5, as well as outer circular pores of varying sizes (with a diameter of 1.4, 0.8, and 0.6 µm, respectively), the manufacturing process must be executed with meticulous precision. To this end, the established ‘laser-assisted precision drawing + post-processing coating’ process, a proven method that has been optimized at critical stages, is to be employed.

Firstly, a customized optical fiber drawing tower is employed, paired with a silicon carbide–quartz composite mold to suppress thermal deformation at high temperatures. During the drawing process, an online laser interferometer meticulously monitors pore ellipticity and size in real time, providing precise morphology data to the traction tension control unit and CO_2_ laser power regulation system. This sophisticated system enables the compression of ellipticity tolerance to a remarkably precise level of ±2.5%, ensuring the meticulous geometric conditions necessary for mode field coupling. This ‘laser-tension synergistic drawing’ process strategy has been consistently applied in the preparation of elliptical microstructure fibers.

Subsequent to the drawing process, a horizontally symmetrical silver coating is applied using plasma-enhanced rotary atomic layer deposition (PE-RALD). A three-dimensional rotating fixture is employed to drive the fiber in a uniform rotation, thereby achieving thickness uniformity of ±1 nm, which is significantly superior to the ±3 nm level of traditional sputtering.

Subsequently, a flat surface is machined in the vertical direction, and angled ion beam sputtering is applied to the machined surface, with real-time monitoring by a crystal oscillator. This process enables the preparation of a dense Si_3_N_4_/TiO_2_/Ag thin film. Finally, the fiber is fixed to a vacuum spin-coating platform, coated with 1 µm PDMS, and the film thickness is measured in real-time using an in situ ellipsometer. Closed-loop adjustment of the rotation speed ensures thickness uniformity within ±5 nm. This process constitutes the entirety of the sensor fabrication process.

## 4. Conclusions

In this study, we propose a two-parameter SPR sensor based on a photonic crystal fiber structure. A strong birefringence effect is achieved through the presence of elliptical pores and a non-rotationally symmetric lattice arrangement. Furthermore, the refractive index and temperature are detected simultaneously. The temperature-sensing channel employs polydimethylsiloxane, a material characterized by a high thermo-optical coefficient. The open channel design ensures rapid interaction with the analyte, thereby enhancing detection efficiency. The orthogonally polarized x and y resonance peaks independently control the RI and temperature measurements, exhibiting a low cross-sensitivity.

The RI-sensing channel demonstrates optimal performance within the 1.33–1.41 range, exhibiting an average wavelength sensitivity of 9400 nm/RIU, a peak response of 18,600 nm/RIU, and a resolution of 5.38 × 10^−6^ RIU. In comparison, the temperature-sensing channel exhibits a maximum sensitivity of 20 nm/°C over the 0–100 °C range, accompanied by a comparable resolution. Discrimination between two parameters, in conjunction with high sensitivity and low crosstalk, renders the PCF-SPR sensor a viable framework for advanced biosensing applications requiring multiparameter analysis.

## Figures and Tables

**Table 1:** Comparison of RI-sensing performance of the proposed biosensor with previous studies.

Structure	Detection Range	Maximum Wavelength Sensitivity	Wavelength Resolution	FOM	Amplitude Sensitivity	Reference
D-shaped/Au/Ta_2_O_5_	1.35–1.46	5000 nm/RIU	2.0 × 10^−5^ RIU	-	266.54 RIU^−1^	[28]
C-groove/Au/TiO_2_	1.37–1.41	29,000 nm/RIU	3.45 × 10^−6^ RIU	-	-	[29]
Gold-coated circular-shaped	1.380–1.401	13,257.20 nm/RIU	-	-	-	[30]
Coated PCF/Au/Ta_2_O_5_	1.25–1.40	21,000 nm/RIU	8 × 10^−6^ RIU	233.33	853.52 RIU^−1^	[31]
Double D-shaped	1.30–1.42	32,100 nm/RIU	-	-	-	[32]
Dual-core/Au/PDMS	1.33–1.42	19,900 nm/RIU	5.03 × 10^−6^ RIU			[33]
D-shaped/Ag/Si_3_N_4_	1.33–1.41	18,600 nm/RIU	5.38 × 10^−6^ RIU	1742.92	10,723.33 RIU^−1^	This work

**Table 2:** Comparison of temperature-sensing performance of the proposed biosensor with other studies.

Structure	Detection Range	Maximum Wavelength Sensitivity	Wavelength Resolution	FOM	Amplitude Sensitivity	Reference
D-shaped/Au/Ta_2_O_5_	−50–50 °C	3.0 nm/°C	3.33 × 10^−2^ °C	-	4.8 × 10^−2^ °C^−1^	[28]
Double D-shaped	0–150 °C	5.9 nm/°C	-	-	-	[32]
Dual-core/Au/PDMS	0–100 °C	8.7 nm/°C	1.13 × 10^−2^ °C	-	-	[33]
Embedded PCF	−20–40 °C	1.1036 nm/°C	-	-	-	[34]
Embedded silver wires PCF	20–320 °C	5.0 nm/°C	2 × 10^−2^ °C	-	-	[35]
Sapphire-based PCF	10–60 °C	12.4 nm/°C	-	-	-	[36]
D-shaped/Ag/Si_3_N_4_	0–100 °C	20.0 nm/°C	5 × 10^−3^ °C	0.27	3.322 °C^−1^	This work

## Data Availability

Data will be made available on request.
